# Interaction between non-coding RNAs, mRNAs and G-quadruplexes

**DOI:** 10.1186/s12935-022-02601-2

**Published:** 2022-04-29

**Authors:** Soudeh Ghafouri-Fard, Atefe Abak, Aria Baniahmad, Bashdar Mahmud Hussen, Mohammad Taheri, Elena Jamali, Marcel E. Dinger

**Affiliations:** 1 Department of Medical Genetics, School of Medicine, Shahid Beheshti University of Medical Sciences, Tehran, Iran; 2 Phytochemistry Research Center, Shahid Beheshti University of Medical Sciences, Tehran, Iran; 3 Institute of Human Genetics, Jena University Hospital, 07740 Jena, Germany; 4 Department of Pharmacognosy, College of Pharmacy, Hawler Medical University, Erbil, Kurdistan Region Iraq; 5 Center of Research and Strategic Studies, Lebanese French University, Erbil, Kurdistan Region Iraq; 6 Skull Base Research Center, Loghman Hakin Hospital, Shahid Beheshti University of Medical Sciences, Tehran, Iran; 7 School of Biotechnology and Biomolecular Sciences, University of New South Wales, Sydney, NSW Australia

**Keywords:** lncRNA, miRNA, mRNA, G-quadruplex, Expression

## Abstract

G-quadruplexes are secondary helical configurations established between guanine-rich nucleic acids. The structure is seen in the promoter regions of numerous genes under certain situations. Predicted G-quadruplex-forming sequences are distributed across the genome in a non-random way. These structures are formed in telomeric regions of the human genome and oncogenic promoter G-rich regions. Identification of mechanisms of regulation of stability of G-quadruplexes has practical significance for understanding the molecular basis of genetic diseases such as cancer. A number of non-coding RNAs such as H19, XIST, FLJ39051 (GSEC), BC200 (BCYRN1), TERRA, pre-miRNA-1229, pre-miRNA-149 and miR-1587 have been found to contain G-quadraplex-forming regions or affect configuration of these structures in target genes. In the current review, we outline the recent research on the interaction between G-quadruplexes and non-coding RNAs, other RNA transcripts and DNA molecules.

## Introduction

G-quadruplexes are secondary helical structures formed as four-stranded nucleic acid structures between guanine (G)-rich nucleic acids. These structures have a helical shape and comprise guanine tetrads that can be made from one [1], two [2] or four molecules [3]. The unimolecular types are usually seen in the telomeric regions, as well as transcriptional regulatory regions [4]. G-quadruplex structures consist of a core G-rich section, including G-tetrads loaded on top of each other and zero or more connecting loops with diverse compositions [5]. The G-rich core classically contains at least two stacked G-tetrads that have a right-handed helical twist. These stacks are combined together by the normal sugar–phosphate backbone. Hydrogen bonds between the Gs in a plane, π–π interactions between the Gs in neighboring surfaces and charge–charge interaction between the partially negative O6 of the G bases and cations provide the binding energy in these structures. Monovalent cations, particularly K+ have the stabilizing role in these structures. Changes of the bases to non-G bases can destabilize these structures [6].

This structure is seen in the promoter regions of numerous genes under certain conditions. G-quadruplexes are involved in several cellular functions, including DNA replication, gene expression, protection of telomeres, and apoptosis [7–9].

These structures reside in important locations in genomic DNA within both coding and non-coding regions. Through residing in these strategic locations, they can participate in several crucial functions at cellular and organismal levels [10]. Formation of these structures between guanine-rich domains renders these regions thermodynamically stable [11]. These structures are formed by telomeric regions in the human genome and oncogenic promoter G-rich regions [12].

DNA G quadruplex-folded regions have been shown to be associated with alterations in transcriptional activity. A recent study has demonstrated that the presence of these structures in promoters is consistently linked with open chromatin configuration and increased transcription of genes. G quadruplex-folded has binding sites of transcription factors activator protein-1 (AP-1) and specificity protein-1 (Sp1), therefore being associated with determination of cell-specific transcriptional programs [13]. A recent study has suggested a model for understanding the mechanism by which G quadruplexes regulate transcription. This model suggests that G quadruplexes act as transcription suppressors through inhibiting polymerase processivity. A bulk of evidence has recently emerged to support this model. However, there is still a misrepresentation of G quadruplexes as transcriptional barriers. In fact, formation of G quadruplexes can potentially affect gene expression at several diverse levels through functioning as an important regulatory mechanism upsetting the landscape of epigenetic marks and chromatin structure [14]. The interaction between formation or disruption of G-quadruplexes and other epigenetic marks such as DNA and histone modifications, nucleosome positioning, and three-dimensional configuration of chromatin has essential role in regulation of gene expression [10].

Notably, wild-type Telomerase reverse transcriptase (hTERT) promoter sequences do not have the ability to make a hairpin structure in solution, yet they can fold into a compact arranged three-G-quadruplex configuration [15]. A number of cancer-related mutations have been shown to destabilize hTERT promoter G-quadruplexes and induce defects in telomere-repeat-binding-factor 2 (TRF2) binding. Notably, ligand-induced stabilization of G-quadruplexes could restore TRF2 binding and hTERT re-inhibition. Cumulatively, these structures are associated with regulation of hTERT activity [16]. Another study has shown that the basic N-terminal Gly/Arg-rich (GAR) domain of TRF2 can bind Telomere-repeat-encoding RNA (TERRA). In fact, disruption of the TERRA-TRF2 GAR complex by small molecules or defects in the GAR domain of TRF2 leads to defects in TERRA, and activation of γH2AX-related DNA damage in telomeres. This could result in reduced telomere length, and induction of telomere abnormalities such as fragility of telomeres. Cumulatively, G-quadruplex structure of TERRA can recognize element for TRF2 GAR domain. Interaction between TRF2 GAR and TERRA has a crucial role in the maintenance of telomere stability [17].

Zheng et al. have shown that an artificial protein can bind G-quadruplexes with high affinity and specificity. This protein has been used to capture G-quadruplexes in living cells from different species, providing the detailed landscape of these structures. This study has shown association between transcription and an extensive construction of G-quadruplexes in genes [18]. Another study has shown direct binding of Yin Yang-1 (YY1) to G-quadruplex structures. Moreover, YY1 binding sites have been shown to have extensive overlap with G-quadruplex structure loci in chromatin. In addition, YY1-mediated long-range DNA looping depends on its dimerization and takes place via its recognition of G-quadruplexes [19].

**Fig. 1:**
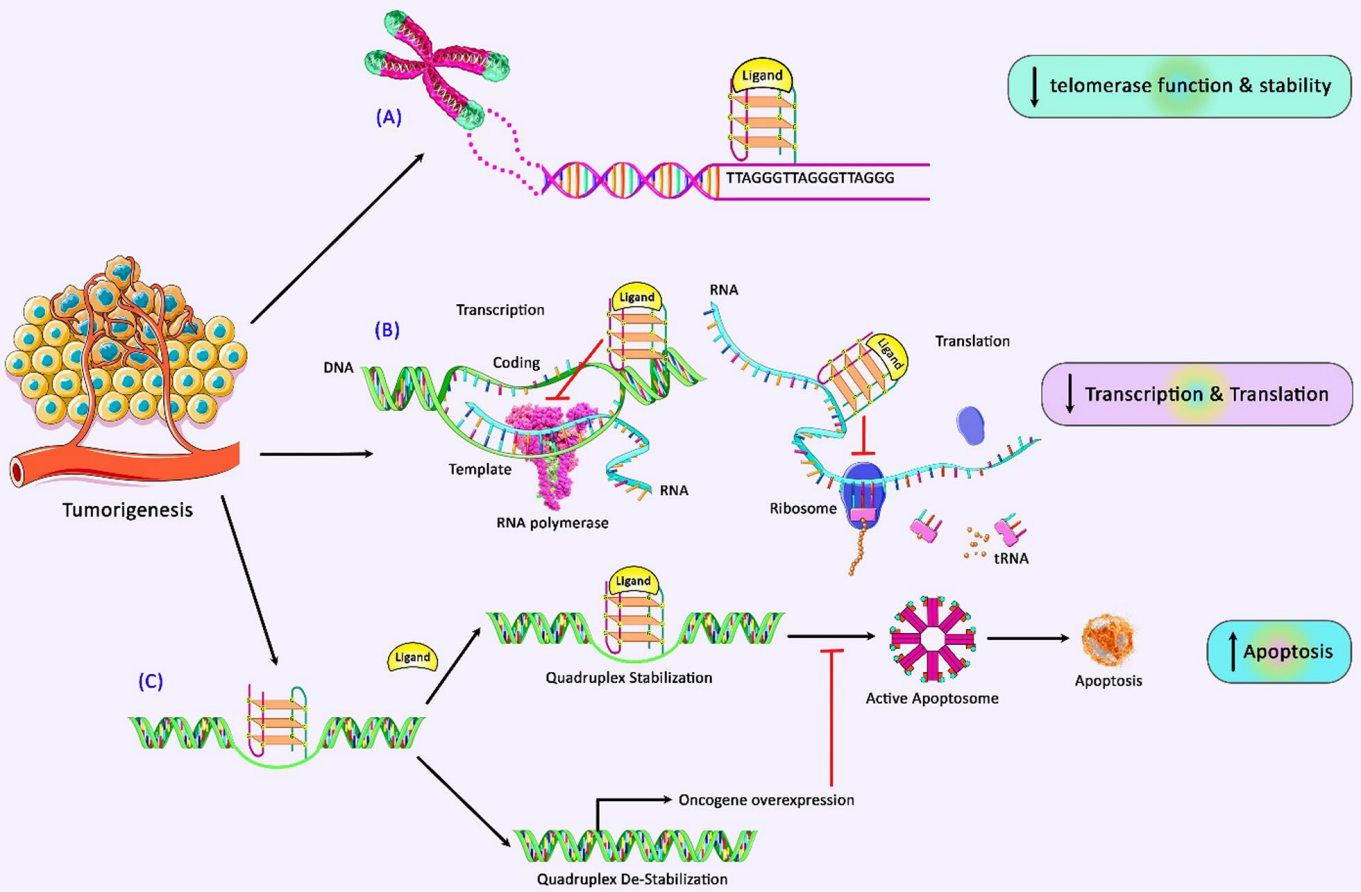
A schematic illustration of the impacts of G-quadruplex ligands on tumorigenesis. Accumulating evidence has illustrated that formation and/or stabilization of G4 structures could play a crucial role as a remedial procedure against cancer cells. Three major remedial methods have been detected recently [23, 24]. Owing to the ligand and cell type G-quadruplex stabilization could result in alterations in **A** interfering with telomere maintenance; G4 formation/stabilization at telomeres was applied as a potential therapeutic tool to suppress telomerase function [25–27], **B** downregulation of oncogenes expression; since most promoters of oncogenes harbor more G4 motifs compared with that of regulatory or tumor suppressor genes, G4 formation could act as a key factor attenuating gene expression of oncogenes [28, 29], **C** activating apoptosis; under particular situations, misregulated G4 structures can cause genome instability, leading to transformation within DNA replication and can trigger DNA damage and recombination events. Increased genome instability results in inducing apoptosis and autophagy in tumor cells [20, 30, 31]

Predicted G-quadruplex-forming sequences are distributed across the genome in a non-random manner. Approximately half of the genes in the human genome are predicted to produce G-quadruplexes near their promoter regions. Many of these structures are present in the vicinity of oncogene promoters or regulatory genes [20]. The transcriptionally active single-stranded form, double-stranded form, and G-quadruplex structures of the promoter are in a delicate balance. Therefore, it is possible to inhibit expression of genes through stabilizing the G-quadruplex structure. Thus, a number of therapeutic strategies have been designed to stabilize secondary DNA structures that are present in the promoter regions of oncogenes. Since stabilization of G-quadruplexes is a possible modality for cancer therapy [21], identification of mechanisms of regulation of stability of G-quadruplexes has practical significance (Fig. 1). In the current review, we outline the recent insights about the presence of G-quadruplexes in non-coding RNAs, other transcripts and DNA molecules.

The G-quadruplex structure within pre-miRNA-149 has been demonstrated to suppress activity of Dicer, therefore decreasing maturation of miRNA-149 in neoplastic cells. Moreover, the inhibitory role of miR-149 on cell proliferation can be achieved through interfering with the G-quadruplex structures existing in the pre-miRNA-149. TmPyP4 (5,10,15,20-Tetrakis-(*N*-methyl-4-pyridyl)porphine) is widely used as a photosensitizer and a modulator of nucleic acid secondary structur with an inhibitory effect on G-quadruplex formation could increase levels of miR-149 in breast cancer cells, leading to reduction of Zinc Finger And BTB Domain Containing 2 (ZBTB2) expression and attenuation of cell proliferation [22].

## Detection of G-quadruplexes in cells and in vitro

Identification of G-quadruplex structures in cells can be accomplished using modified antibodies, G-quadruplex based aptamers as well as small molecules [32]. G-quadruplexes can be detected in chemically fixed human cells using specific antibodies. Previous studies have used immunostaining [33] or immunoprecipitation [34] techniques for this purpose. In these techniques, antibodies against G-quadruplexes bind with these structures in a non-native cellular setting following some treatments. However, these treatment steps might affect construction of G-quadruplex. It has been demonstrated that treatment with RNAse H can remove G-quadruplex in the non-transcribed strand of R-loop [35]. Therefore, identification of G-quadruplexes in living cells is a preferred method. Since antibodies cannot permeate into cells, it is not possible to use them for living cells. Moreover, cytoplasm has a reducing environment which interferes with construction of the disulfide bonds needed for preserving the tertiary structure of antibodies [36]. Zheng et al. have reported an artificial G-quadruplex probe protein which specifically binds G-quadruplex with high affinity. Application of this probe in living cells has led to identification of genome-wide landscape of G-quadruplexes [37].

## Non-coding RNAs and G-quadruplexes

Both long non-coding RNAs (lncRNAs) and microRNAs (miRNAs) have been found to contain G-rich regions predicted to form G-quadruplexes. For instance, *NEAT1* contains such regions. *NEAT1* functions as a platform for the assembly of paraspeckles, which are a type of organelle within the nucleus participating in the regulation of gene expression. Assembly of paraspeckles requires *NEAT1*-mediated recruitment of the RNA-binding protein NONO. NONO has been found to bind with the G-quadruplex structure of G-rich C9orf72 repeat RNA. *NEAT1* contains several G-quadruplex motifs. Furthermore, NONO can bind with *NEAT1* G-quadruplexes in a specific manner suggesting that G-quadruplex motifs may facilitate interaction between NONO and NEAT1. Notably, NONO binding sites on *NEAT1* mainly correspond to G-quadruplex motifs. Consistent with this finding, disruption of G-quadruplexes with specific small molecules leads to separation of intuitive NONO-*NEAT1* complexes. Therefore, G-quadruplexes have been suggested as principal candidates for the NONO-recruiting components of *NEAT1* [38].

Another study has reported the presence of an evolutionarily conserved G-rich region at the 5′ end of the *H19* coding gene which is predicted to form a G-quadruplex structure. Further assays with G-quadruplex-specific ligands have shown the ability of the G-rich motif adjacent to the transcription start site in constructing a G-quadruplex. Most notably, this G-quadruplex has been found to regulate expression of the *H19* gene. Sp1 and E2F1 are two transcription factors that interact with this G-quadruplex to either inhibit or enhance transcription of *H19*, respectively. In addition, expression of *H19* in the course of differentiation of mice embryonic stem cells seems to be controlled by a G-quadruplex structure in this lncRNA [39].

Telomeric Repeat-containing RNA (TERRA), which is transcribed from telomeres, has been shown to contain G-quadruplex structures. Notably, unstructured hinge domains that participate in the targeting of HP1α to constitutive heterochromatin regions recognize these structures of TERRA. Through this mechanism, TERRA contributes to the enrichment of HP1α at telomeric regions to preserve the heterochromatin structure. Additionally, HP1α has been shown to bind more quickly to G-quadruplex structures having parallel topology versus those with antiparallel topology. These G-quadruplex structures have been seen in the regulatory portions of numerous oncogenes. Therefore, such non-canonical configurations have been determined as regulators of HP1α function and chromatin domain structure participating in the epigenetic regulation of gene expression [40].

In an attempt to assess the expression profile of colon cancer samples, Matsumura et al. have identified a novel up-regulated lncRNA in colon cancer tissues, namely FLJ39051 or GSEC (G-quadruplex-forming sequence containing lncRNA). Inhibition of GSEC lncRNA transcription resulted in a significant decrease in the motility of cancer cells. This lncRNA was shown to bind to the DHX36 RNA helicase through its G-quadruplex-forming sequence and suppress the DHX36 G-quadruplex unwinding function. Thus, GSEC is thought to participate in the migration of colon cancer cells through suppression of activity of DHX36 through its G-quadruplex configuration [41]. Interestingly, G-quadruplex structures are conserved in L1 sub-families and may therefore result in an increased level of retro-transposition activity [42].

In addition to these G-quadruplex-containing lncRNAs, some lncRNAs have been found to alter expression of genes by affecting G-quadruplex structures in target genes. LncRNA *XIST* has been found to be processed into a small transcript called XPi2. This small transcript has a gender-independent expression pattern, implying that it has a role outside of X-chromosome inactivation. Nucleolin and hnRNP A1 are two XPi2-related proteins that are functionally associated with G-quadruplex structures. XPi2 silencing has decreased the activity of the KRAS pathway. Further studies have supported the interaction between XPi2 and the polypurine–polypyrimidine regions of KRAS. Therefore, XPi2 might induce expression of KRAS by decreasing G-quadruplex formation. Thus, it suggests that XPi2 has a role in KRAS- associated tumorigenesis through regulation of G-quadruplex structures [43].

A further group of lncRNAs has neither G-quadraplex structures nor directly regulate expression of target genes through affecting stability of these structures, but instead have interactions with ATP-dependent RNA helicases such as RHAU that exhibit high affinity for G-quadruplex structures. An example of these lncRNAs is *BC200*. This lncRNA does neither form G-quadruplex structures nor interact with the quadruplex-interacting region of RHAU, but it directly binds to RHAU. *BC200* acts as an acceptor of unwound quadruplexes through a cytosine-rich area in the vicinity of the 3′-end of this transcript. *BC200* has an interaction with the G-quadruplex-containing telomerase RNA. Therefore, RHAU might facilitate BC200 binding and consequent regulatory effects with G-quadruplex-harboring nucleic acid regions [44].

**Fig. 2:**
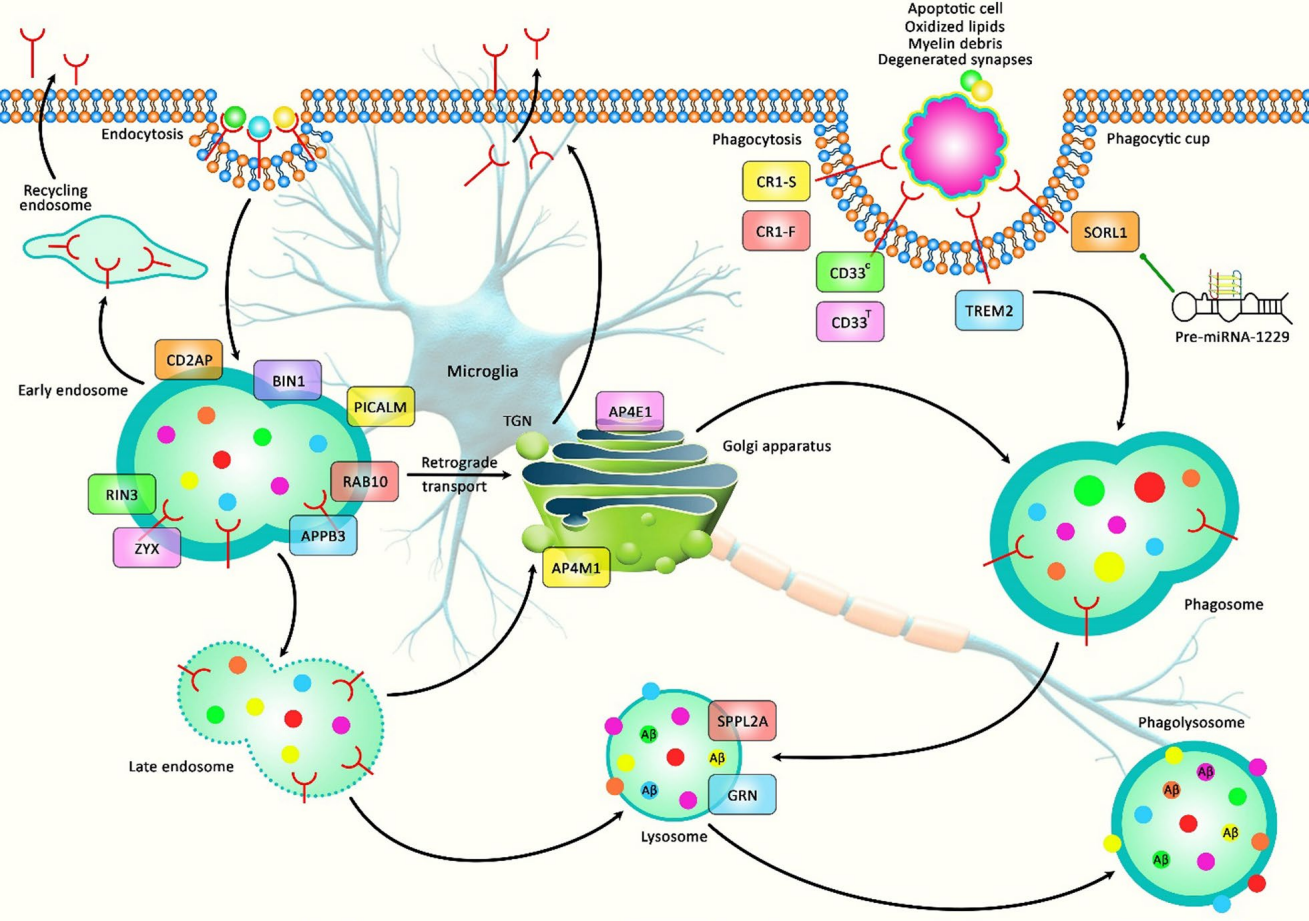
A schematic representation of the interaction between G-quadruplex structure of pre-miRNA-1229 and endolysosomal processing in microglial cells in Alzheimer’s disease. Previous studies have authenticated that SORL1 could play an effective role in binding Aβ and facilitating its degradation in the lysosomes [46]. As an illustration, recent study has detected that Pre-miRNA-1229 rs2291418 variant, which is significantly associated with Alzheimer’s diseases, alters its structure to the extended hairpin structure and enhances SORL1 transcription. SORL1 is a protein that is responsible for the processing and trafficking of Aβ in AD [45]

Importantly, G-quadruplexes within premature microRNA precursors (pre-miRNAs) might also affect risk of human disorders. For instance, pre-miRNA-1229 has been shown to contain a variant that is associated with risk of Alzheimer’s disease. The mature form of this miRNA modulates translation of SORL1. Pre-miRNA-1229 forms a G-quadruplex configuration that co-occurs in a balanced state with the canonical hairpin structures, possibly regulating levels of miR-1229-3p. Most notably, the Alzheimer’s disease-associated variant of pre-miR-1229 appears to alter the balance between these structures. Consequently, this G-quadruplex structure has been suggested as a putative therapeutic target for Alzheimer’s disease [45] (Fig. 2).

The G-quadruplex structure within pre-miRNA-149 has been demonstrated to suppress activity of Dicer, therefore decreasing maturation of miRNA-149 in neoplastic cells. Moreover, the inhibitory role of miR-149 on cell proliferation can be achieved through interfering with the G-quadruplex structures existing in the pre-miRNA-149. TmPyP4 with an inhibitory effect on G-quadruplex formation could increase levels of miR-149 in breast cancer cells, leading to reduction of ZBTB2 expression and attenuation of cell proliferation [22].

In a separate example involving G-quadruplexes and miRNAs, miR-1587 has been shown to contain a G-rich region forming stable G-quadruplex structure in certain conditions described by the presence of potassium and sodium ions and low levels of ammonium cation. High levels of ammonium cation or molecular crowding milieus have been shown to induce a dimeric G-quadruplex in miR-1587 via 3′-to-3′ assembling of two monomeric G-quadruplex units with one ammonium ion confined between the edges. Notably, two manufactured jatrorrhizine products have been shown to trigger the dimerization of miR-1587 G-quadruplexes. On the other hand, jatrorrhizine could not induce this structure in spite of its ability to bind with the dimeric miR-1587 G-quadruplexes [47].

Table 1 shows the list of noncoding RNAs which have G-quadruplexes or regulate expression of genes through modulation of these structures.

**Table 1:** The presence and function of G-quadruplexes in non-coding RNAs

Non-coding RNAs	Location	Ligands	Cell line	Clinical samples/animal models	References
*NEAT1*	–	NONO	HEK293T	–	[38]
*H19*	5′ end	Sp1, E2F1	HEK293T, HeLa, U2OS, EpH4, mESCs	–	[39]
*XIST*	–	Nucleolin, hnRNP A1, KRAS	Huh-7, MCF-7, normal lymphoid cells	31 colon cancer tissues and their adjacent non-tumorous tissues	[43]
*FLJ39051 (GSEC)*	–	DHX36	DLD-1, SW480	105 samples of colon Carcinoma tissues and normal colon samples	[41]
*BC200 (BCYRN1)*	–	RHAU	HEK293T, HeLa, MCF-7, T47D, MDA-MB-231, SK-BR-3, A549	–	[44]
*TERRA*	–	HP1α	NIH3T3	–	[40]
*pre-miRNA-1229*	–	SORL1	–	–	[45]
*pre-miRNA-149*	–	ZBTB2, Porphyrin	MCF-7	–	[22]
*miR-1587*	3′-UTR	TAGLN, pseudopalmatine, TMPyP4	HeLa	–	[47]
*pre-miRNA 92b*	–	LNA	A549 cells, NSCLC	–	[48]
*LINE-1*	3′-UTR	–	HeLa	–	[42]
*miR-3620-5p*	5′ and 3′ ends	Sanguinarine	–	–	[49]
*pre-let-7*	–	Lin28	Human NCCIT embryonal carcinoma cells	–	[50]

## Protein coding transcripts and G-quadruplexes

G-quadruplexes structures are enriched in 3′ UTRs of mRNAs, where miRNAs could also bind. G-quadruplex structures have been shown to affect miRNA binding to target mRNAs, providing a new mechanism for G-quadruplex-dependent modulation of miRNA-mRNA interactions that have fundamental roles in the maintenance of gene expression [51]. FMRP binding with the G-rich region of the PSD-95 transcript provides an important example of the interference of G-quadruplexes with miRNA-mRNA interactions. Notably, 3′ UTR of PSD-95 transcript has a miR-125a binding site which is located in a G-rich region bound by FMRP. FMRP regulates expression of PSD-95 through phosphorylation-related mechanisms. Both unphosphorylated FMRP and its phosphomimic FMRP S500D have a high affinity for binding with G-quadruplexes within PSD-95 transcript. However, only FMRP S500D can bind with to miR-125a. Therefore, FMRP functions as a molecular switch to regulate stability of the complex constructed between the miR-125a-RISC and PSD-95 transcript in a phosphorylation-dependent manner [52].

G-quadruplexes have also been found to form within other regions of mRNAs. For instance, a G-quadruplex structure has been found within the 5′ UTR of the potassium 2-pore domain leak channel *Task3* transcript. The stability of this structure has been preserved under physiological ionic concentrations. Such structures can inhibit translation of *Task3*. A G-quadruplex-specific helicase, namely DHX36 can intervene with this structure resulting in as increase in K+ leak flow and induction of membranes hyperpolarization. The G-quadruplex structure has an essential role in transport of *Task3* transcripts to distal primary cortical neurites. Since abnormal Task3 levels have been correlated with abnormal function of neurons, the role of this G-quadruplex in the regulation of K+ leak within neurons shows the importance of this route in the pathogenesis of neurological disorders [53].

*BAG-1* is another gene that contains a G-rich region in its 5′UTR capable of forming a G-quadruplex structure. This structure has been found to regulate cap-dependent as well as cap-independent expression of *BAG-1*. SNRPA has been demonstrated to bind directly to the BAG-1 transcript via these G-quadruplexes modulating expression of the BAG-1 gene [54].

Another study has shown that translation of NRXN2α is regulated by a G-quadruplex structure in its 5′ UTR. The 5′ UTR of this gene has an inhibitory effect on its expression. Notably, there is a crucial subregion in this area that accounts for the main inhibitory effect through formation of a certain secondary G-quadruplex configuration. In addition, the upstream AUGs work in a synergistic mode with G-quadruplex to inhibit NRXN2α-expression. Therefore, the 5′ UTR of *NRXN2α* suppresses its translation through different mechanisms [55].

**Fig. 3:**
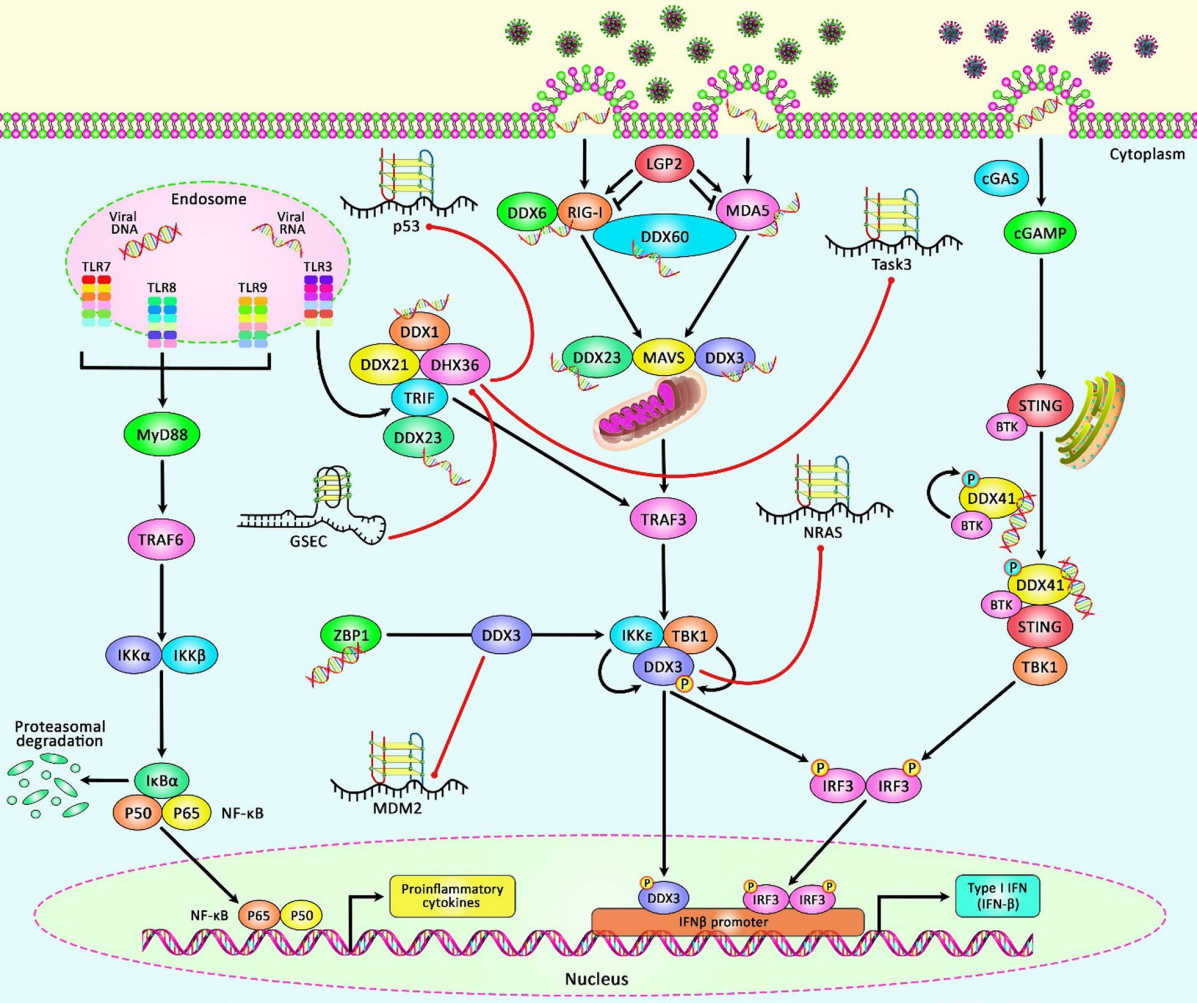
A schematic diagram of the interaction between lncRNA, mRNAs and G-quadruplexes in modulating DEAD-box helicases in antiviral innate immunity signaling cascades. DEAD-box helicases have been detected to have a significant role as sensors for nucleic acids, containing dsRNA, cytoplasmic DNA, and viral RNAs, resulting in triggering the induction of interferon and interferon-stimulated genes. Besides, DEAD-box helicases could involve in innate immune cascade downstream of nucleic acid-sensing via modulating protein-protein interactions and elevating the DAMP cascade [48–50]. Previous studies have authenticated the interaction between ncRNAs, mRNAs and G-quadruplexes regulating DEAD-box helicases members containing DDX3 and DHX36. As an illustration, accumulating finding has suggested that lncRNA GSEC could play a crucial role in colon cancer cell migration via suppressing the function of DHX36 through its G-quadruplex structure. In fact, GSEC could bind to DHX36 RNA helicase via its G-quadruplex-forming sequence, thereby attenuating DHX36 G-quadruplex unwinding activity [9]. In addition, another research has demonstrated that DHX36 could bind to the p53 RNA G4-forming sequence, whilst a mutation in the p53 G4 sequence could result in suppressing the DHX36 function to process its pre-mRNA 3′-end [34]. All the information regarding the presence and function of G-quadruplexes in ncRNAs and mRNA coding genes can be seen in Tables 1 and 2.

In spite of the observed inhibitory effect of G-quadruplexes in the 5′ UTR of genes on their expression [55], two G-quadruplex structures in the 5′ UTR of *TGFβ2* mRNA have been shown to increase its expression. These structures are thought to work additively to noticeably upregulate *TGFβ2* expression [56]. Consistent with this study, is the enhanced expression of an isoform of ARC2 through the presence of a G-quadruplex structure in the longer variant of the 5′ UTR of the *ARPC2* transcript. This variant of the 5′ UTR also has an internal ribosome entry site (IRES). This variant of 5′ UTR has been shown to promote cap-independent translation of ARPC2. The G-quadruplex also contributes to the activity of IRES. Consistent with the supposed role of IRES in induction of expression of genes under cellular stress through cap-dependent translation, expression of *ARPC2* has been shown to be increased at high cell density. Therefore, it is proposed that a mechanistic model of IRES upregulation is underpinned by the G-quadruplex motif exposed from the chief stem-loop component [57]. Figure 3 demonstrates the interaction between lncRNA, mRNAs and G-quadruplexes in the regulation of DEAD-box helicases in antiviral innate immunity signaling pathways (Table 2).

**Table 2:** The presence and function of G-quadruplexes in mRNA coding genes

Protein-coding genes	Location	Ligands	Cell line	Clinical samples/animal models	References
*Task3*	5′-UTR	DHX36	HEK293	C57Bl/6 wild-type mice	[53]
*BAG-1*	5′-UTR	SNRPA	HCT-116	–	[54]
*NRXN2α*	5′-UTR	–	SH-SY5Y, SK-N-SH, U87MG, HEK293	–	[55]
*TGFβ2*	5′-UTR	–	MCF-7	–	[56]
*ARPC2*	5′-UTR	–	HEK293, MCF7	–	[57]
*KNCJ11*	3′-UTR	miR-331-3p, TMPyP4	HEK-293A	Cardiomyocytes isolated from newborn SD rats	[51]
*PSD-95*	3′-UTR	FMRP, miR-125a	–	–	[52]
*NRAS*	5′-UTR	ZnAPC	MCF-7	–	[58]
	5′-UTR	–	DDX3X, DDX5, DDX17, GRSF1, NSUN5	HeLa, Flp-In T-REx 293	[59]
*Hnf4a1*	5′-UTR	–	HEK293T, HepG2-C3A	–	[60]
*Bcl‐2*	5′-UTR	–	HEK293, A‐375	–	[61]
*SMNDC1*	5′-UTR	FMRP	–	E17 mouse brain lysate	[62]
*FADS2*	3′-UTR	mir331-3p	HEK293, Huh7, HCT116	–	[63]
*PITX1*	3′-UTR	RHAU	–	–	[64]
*p53*	3′-end	DHX36	A549, HCT116 WT, p53 KO cells	–	[65]
*SNCA*	5′-UTR	–	HEK-293, SK-N-SH, Neuro-2a	–	[66]
*NRF2*	5′-UTR	EF1a	HEK293, HeLa	–	[67]
*ADAM10*	5′-UTR	A methyl-quinolinium derivative	HeLa, HEK-APP	–	[68]
*VEGF*	5′-UTR	360A, Phen-DC	HeLa	–	[69]
*NR2B*	3′-UTR	FMRP	–	E17 mouse brain lysate	[70]
*Nkx2-5*	5′-UTR	RHAU	H9C2, 293T, COS7, E14.5 cardiomyocytes	Rhau conditional KO mice	[71]
*APP*	3′-UTR	–	HeLa, HEK293	–	[72]
*Gap-43*	5′-UTR	hnRNP-Q1	N2a cells	Timed pregnant C57BL/6J mice	[73]
*MDM2*	Adjacent to an E-box DNA motif	ACBL, hnRNP family, XRCC6, XRCC5, DDX3X, EWS, Ku70-Ku80 protein dimer	93T449, HNLF, WDLPS	–	[74]
*PIM1*	–	–	–	–	[75]
*c-MYC*	Promoter	A quinoxaline analog: 6,7-Difluoro-2,3-bis(4-(4-methylpiperazin-1-yl) phenyl) quinoxaline (QN-1)	4T1 cells	Female BALB/c mice	[76]
*HOXC10*	–	CHD7	HeLa, MDA-MB-231	–	[77]
*CD44*	Splicing cis element	hnRNPF	HMLE, HMLE-Twist-ER, MCF10A, HEK293	–	[78]

## Discussion

Several lncRNAs and mRNA coding genes have been found to contain G-quadruplexes or regulate expression of genes through modulation of these structures. G-quadruplexes have prominent roles in the regulation of gene expression and interventions with these structures are emerging as novel therapeutic opportunities in genetic diseases such as cancer. Therefore, identification of lncRNAs/mRNAs that modulate G-quadruplexes is important.

G-quadruplexes are also being discovered in miRNA precursors suggesting a role for RNA G-quadruplexes in the regulation of miRNA biogenesis and control of interaction of non-coding RNAs with their partners [79].

Notably, numerous identified G-quadruplexes in non-coding RNAs has been shown to be unstable, being detected only in the presence of certain ligands or ions [79]. Moreover, G-quadruplexes in pri-miRNAs and pre-miRNAs are present in a well-regulated balance with the hairpin structure. This balance can permit appropriate regulation of gene expression [79].

Secondary structures formed within non-coding RNAs can determine their mode of action and their effect on targets of these transcripts. Notably, the G-quadruplex structures within precursor miRNAs can affect the structure of hairpin stem loops that have essential roles in recognition of these structures by Dicer recognition and additional maturation steps [22]. The importance of this finding is further highlighted by the observation that approximately 16% of identified human pre-miRNAs can embrace G-quadruplex structures instead of acknowledged stem-loops [80]. Consistently, a number of studies have suggested stimulation of G-quadruplex development and dimerization as a new approach for regulation of functions of certain miRNAs [47]. Meanwhile, G-quadruplex structures exist in the 3′ UTR of mRNAs, a region which is targeted by miRNAs. Therefore, G-quadruplexes can affect miRNA-mRNA interactions.

Among G-quadruplex-containing lncRNAs are those with significant roles in carcinogenesis, such as *NEAT1*, *H19* and *GSEC*, providing additional evidence for association between these structures and malignant transformation of cells. In addition, G-quadruplexes within non-coding RNAs might affect the pathogenesis of neurologic disorders such as Alzheimer’s disease [45].

## Conclusions

Taken together, we have summarized evidence for prevalent presence of G-quadruplexe structures in both miRNAs and lncRNAs and association between construction of these structures within pri- and pre-miRNAs and miRNA synthesis. Moreover, G-quadruplexes have been found to affect binding of miRNAs and lncRNAs with their target transcripts and interacting proteins, respectively.

G-quadruplexes represent novel targets for therapeutic interventions. As some transcripts can alter these structures, manipulation of expression of these transcripts is a possible therapeutic option in this regard. Meanwhile, G-quadruplex structures have been found in several disease-associated lncRNAs and miRNAs. Thus, alteration in the stability of G-quadruplexes within these transcripts is a possible mechanism for dysregulation of these transcripts in human disorders, such as cancer and other genetic diseases.

Since the presence of these structures in RNA molecules might participate in the mechanisms that pathogenic organisms or tumors exploit to evade the hosts’ immune responses, future research in this field would pave the way for identification of novel strategies to combat these two kinds of disorders.

## Data Availability

The analyzed data sets generated during the study are available from the corresponding author on reasonable request.
